# Ramadan-specific nutrition education improves cardio-metabolic health and inflammation—a prospective nutrition intervention study from Pakistan

**DOI:** 10.3389/fnut.2023.1204883

**Published:** 2023-12-22

**Authors:** Rahmat Gul, Imran Khan, Iftikhar Alam, Ali Almajwal, Imtiaz Hussain, Namrah Sohail, Muhammad Hussain, Hellas Cena, Sunara Shafiq, Anam Aftab

**Affiliations:** ^1^Department of Human Nutrition, Faculty of Nutrition Sciences, The University of Agriculture, Peshawar, Pakistan; ^2^Department of Dietetics and Nutritional Science, Faculty of Pharmacy and Allied Health Sciences, University of Sialkot, Sialkot, Pakistan; ^3^Department of Human Nutrition and Dietetics, Bacha Khan University, Charsadda, Pakistan; ^4^Department of Community Health Sciences, College of Applied Medical Sciences, King Saud University, Riyadh, Saudi Arabia; ^5^Department of Food Science and Technology, University of Poonch Rawalakot Azad Jammu and Kashmir, Poonch, Pakistan; ^6^Dietetics and Clinical Nutrition Laboratory, Department of Public Health, Experimental and Forensic Medicine, University of Pavia, Pavia, Italy; ^7^Department of Dietetics and Nutritional Sciences, University of Sialkot, Sialkot, Pakistan

**Keywords:** DEAR, PDGN, inflammatory cytokine, CRP, Ramadan fasting, NEAT

## Abstract

There are recent reports that Ramadan fasting (RF) results in weight gain instead of weight loss. In addition, the data on the efficacy of brief nutrition education on healthy eating practices in Ramadan for better health are scarce. Therefore, a study was conducted to investigate the effects of brief nutrition education before the start of RF on healthy eating practices during RF. For this purpose, a prospective observational study focused on “Dietary Education and Awareness for Ramadan (DEAR)” as an intervention was carried out. The participants (*n* = 74) were recruited and divided into two groups, i.e., intervention and control groups (*n* = 37 each). As an intervention, nutrition education lessons were given before and during RF month. The control group did not attend these nutrition education lessons. Data on anthropometrics, dietary intake, and other parameters were collected at three time points: before, in the end, and 4 weeks after RF. Weight was measured in kg; height, waist circumference (WC), and hip circumference (HC) were measured in cm; and body mass index (BMI) was calculated. Waist-to-hip ratio (WHR) was calculated by dividing the waist value by the hip value. Body composition analysis was performed by the body composition analyzer (BF-907). Blood pressure (BP) was measured using a validated automated blood pressure. A 3–5 ml of venous blood was collected, and plasma and serum were separated. Serum and plasma samples were processed for general blood chemistry (blood lipid profile, glucose, and CRP) within 2 h. CRP was determined by the immunoturbidimetry method using an auto-analyzer. An enzyme-linked immunosorbent assay (ELISA) was used to determine cytokine/chemokines. Adherence to nutrition education (intervention) was assessed. The results show that nutrition education has positive effects on overall nutrition. Significant improvement in dietary adherence to dietary advice in the intervention group was noted. Significant BW loss (mean loss: 1.21 kg) in the intervention group was observed. The majority (63.3%) had lost BW ≥ 1.0 kg. Other changes observed as a result of the intervention included improvements in blood glucose, cholesterol, CRP levels, and systolic and diastolic BP. There was a notable shift in pro- and anti-inflammatory cytokine concentrations: IL-7, IL-4, and TGF-α decreased, while IL-2, TNF-α and resistin, IL-1 RA, IL-17 A, and sCD40 increased. In conclusion, RF resulted in a loss in mean BW and an improvement in related blood chemistry and cytokine profiles. Furthermore, nutrition education before RF resulted in better nutrition practices during RF and a desirable healthy BW, blood lipid, and cytokine profiles.

## Background

Ramadan is the ninth month of the Islamic Calendar. Muslims all over the world observe fasting during this month from dawn to sunset. During fasting hours, Muslims refrain from fluid and food intake (1, 2). The duration of fasting hours is variable and ranges from 12 to 18 h, depending on the season and geographical location (3). The prolonged daytime fasting during Ramadan makes it a unique model of weight management strategy. Furthermore, during this month, changes in a number of dietary habits occur. These changes include quality, quantity, timing of food intake, physiological, and biochemical factors (4, 5). These changes may be helpful in reducing the prevalence of certain chronic disorders, such as obesity, diabetes, and cardiovascular diseases.

Obesity, a chronic inflammation, is globally prevalent at alarming rates. Obesity is indisputably related to an increased prevalence of many preventable diseases, a significant log-linear increase in the risk of all-cause mortality, an increasing body mass index (BMI) value of >25, and enormous economic losses. While a number of strategies have been in place in an attempt to minimize the undesirable changes in metabolism and appetite, the benefits of an intermittent fasting regimen have begun to demonstrate strong efficacy in studies including ours (6) and others (7–9). A change in body weight is associated with other changes in the body, for example, changes in body composition, blood biochemistry, and a number of inflammatory biomarkers. Extended durable changes in the circadian clock of the human body may encourage numerous changes in cardio-metabolic profile; thus, fasting during Ramadan is well-established to have an overall impact on endocrine and cardio-metabolic health (7–9).

A number of classical prospective epidemiological studies have reported a constant relationship between high CRP levels and an increased possibility of cardiovascular episodes comprising myocardial infarction, stroke, and cardiovascular disease (CVD)-related mortality (10–14). Other markers that are related to obesity and cardiovascular health include cytokines (pro- and anti-inflammatory) and chemokines. Although the effects of short-term fasting on the profile of inflammatory markers have also been previously documented (15), the effect of long-lasting variations of food consumption in a model such as Ramadan fasting on the markers of inflammation has not been extensively studied. Nevertheless, the control diet intake has been recognized as an effective mechanism for managing obesity (16).

For most individuals, diet control may be persuaded by nutrition education; the education may, therefore, possibly aid the improvement of dietary interventions. Interestingly, controlling excessive food intake is a commendable act in Islamic teachings and health guidelines (17, 18). Faith-based interventions such as the dietary interventions adopted by Christians have also been reported to be fruitful in managing weight and its associated health risks (19, 20). Ramadan Iftar buffet, for example, is a common tradition now throughout the Muslim world (21), and the common belief among Muslims is that Ramadan is a month of ‘giving charities’ and ‘generosity’ (22). It is obvious that there is a gap between thoughtful knowledge and attention in rationalizing feasting and expenditure. The gap needs to be spanned by more nutrition/behavior sensitive education programs specially premeditated for the local public about the concept that Ramadan is also a month of modesty—modesty in food intake as well. There is a dire need for nutrition educationalists and researchers to be involved in augmenting the gap—a notion supported by many researchers. As Ramadan fasting is a natural way of observing dietary restrictions and neither exaggerate nor extremely relaxes dietary and lifestyle changes, any nutrition education is supposed to be very successful.

In addition, despite the role of Ramadan-specific nutrition education, it has been extensively studied in metabolic disorders patients; however, no studies, to the best of our knowledge, have been reported in healthy individuals. Having in mind that the observed variation in changes in body weight and body composition may be a result of Ramadan fasting, we hypothesize that fasting with some advice on healthy dietary intake may have an impact on overall health. We were of the view that any healthy dietary modification is deemed to be easy to take place as well as to be adhered to during the post-Ramadan period as well. We looked to create long-term dietary behavioral changes as proposed elsewhere (23, 24) and learned during the month of Ramadan through developing practical skills that help in healthy dietary intake.

We, therefore, conducted this study to investigate any long-term positive effects of Ramadan fasting through a follow-up post-Ramadan phase to observe adherence to healthy dietary and lifestyle modifications brought about during Ramadan. Furthermore, we wanted to observe the efficiency of nutrition education-based dietary interventions to maneuver post-Ramadan weight regain. We expected that the intervention would help in encouraging participants to keep track of their food intake at feasting times during Ramadan fasting. It was hypothesized that the control food intake and the detainment of Ramadan-induced weight loss would be more obvious in the nutrition intervention group than in the control group. In order to investigate the rationale of how fasting during Ramadan affects the body weight and body composition of practicing Muslims, it might lead to a better interpretation of the global practicing Muslim community being influenced by observing the religious obligations of Ramadan. Furthermore, it might be valuable to take into account the epidemic of overweight and obesity, which has affected nations around the world, including Muslim-majority countries (25).

## Materials and methods

The study used a pre- and post-test design with random assignments to the intervention group, who received special structured nutrition education through structured lectures, individual telephone counseling, and other educational tools. The control group did not receive any specially structured nutrition education lessons. Both groups completed pre- and post-test as reported elsewhere (6).

### Recruitment of study participants

The participants of this study were either members of a registered organization, i.e., nutrition education, awareness, and training (NEAT: Social Welfare Department Khyber Pakhtunkhwa, Govt. of Pakistan) or persons known to members of the organizations. Potential participants were identified from the member registration record of NEAT. The registered members were encouraged to invite their other family members, relatives, and friends to join a pre-education session 1 week before the start of Ramadan fasting month in 2018.

On their arrival, the anthropometrics and body compositions of all participants were measured. Participants were encouraged to provide their willingness to participate as participants in the control group of the study or the nutrition intervention group. Initially, 37 male participants showed their interest in participating in the nutrition intervention group. These were enrolled as ‘the intervention group’. A group of *n* = 37 perfectly matched those in the intervention group with respect to their baseline education, age, weight, and BMI was to act as the control group.

### Inclusion and exclusion criteria

The detailed inclusion and exclusion criteria have been reported previously (6), but briefly, the study excluded bedridden and sick individuals, antenatal and breastfeeding females, and patients with conditions that necessitate dietary adjustments. Participants with pre-diabetes symptoms as monitored by a medical doctor were also not included. All participants were screened for their general health by a medical doctor before they entered the study. The research team had access to the personal health files of the selected participants as most of them were members of NEAT and were routinely checked for their health status from time to time. There were no refusals among the respondents who were chosen.

### Intervention

The intervention was in the form of brief sessions of nutrition education. Nutrition education was primarily based on a set of dietary advice for Ramadan fasting, specifically developed for the purpose of the present study, i.e., Dietary Education and Awareness for Ramadan (DEAR; Supplementary File 1). These advices were prepared using information from Pakistan Dietary Guidelines for better nutrition (PDGN). The nutritional recommendations of the World Health Organization were also incorporated into these guidelines (26).

### Data collection

All data were collected at three time points (T1, T2, and T3; repeated measures). Data at T1 were collected 2–3 days before the start of the fasting month of Ramadan. Data at T2 were collected in the last week of Ramadan fasting, preferably 2–3 days before the end of Ramadan fasting. Data at T3 were collected 1 month after Ramadan fasting. To ensure relatively huge data in a comparatively short time span, the help of trained field and clinical data collectors was sought.

### Dietary data

The participant’s dietary data were collected using a 3-day-24 HDR and a Food Frequency Questionnaire (FFQ; 27). The FFQ was specially developed for this research. Dietary recalls were conducted in the form of face-to-face interview. During the interviews, participants were asked to recall their food intake during the fasting period in a certain order, starting with breakfast in the morning (Sahar meal), Iftar meal, and ending with their last meal before sleep. Data on cooking methods (roasted, fried, boiled, steamed, etc.) and food sources (homemade vs. outsourced) were also gathered. Meanwhile, a family adult confirmed the nutritional report in interviews to reduce misinterpretations. Models of household utensils, such as bowls, spoons, and cups, were utilized to aid in the measurement of food consumption.

Amounts were reported based on how much food was consumed from each bowl, i.e., a half-filled small bowl. Respondents were asked to cup equals when they gave an unclear answer (e.g., “I used a little or a lot of milk in tea”). Food portion sizes were calculated using data from Pakistan’s Dietary Guidelines for Better Nutrition (28).

The average amount of each food item consumed for 3 days was determined, and nutrients were calculated. Nutrient intake was calculated using an in-house nutrient calculator based on the data from Pakistan’s food composition tables and previously published research (6, 29). Carbohydrates, lipids, protein, total energy, and a few vitamins and minerals were among the nutrients examined. There were additional percentages of energy from carbohydrates, lipids, and protein. Given the importance of energy distribution in solid vs. liquid foods (30), we also calculated the percentage of energy contribution by solid and liquid diets to assess the caloric intake in terms of ‘solid calories’ and ‘liquid calories’. Participants were also requested to disclose all types of prescribed medicines and supplements consumed.

### Anthropometric measurements

Weight was measured in kg, and height, waist circumference (WC), and hip circumference (HC) were measured in cm, as previously reported (6). In brief, body mass index (BMI) was calculated by dividing weight by squared height. The waist circumference was recorded at the height of the navel, and the hip circumference was measured at the crest of the buttocks by using a non-stretchable measuring tape. Waist-to-hip ratio (WHR) was calculated. Participants were categorized as obese (OB), overweight (OW), and normal weight (NW) based on their BMI values according to WHO standards as previously reported (6).

### Assessment of body composition

Body composition analysis of the study participants was performed using a body composition analyzer (BF-907) as reported previously (6). It is portable, non-invasive battery-operated equipment connected to the body through four electrodes.

### Blood pressure measurement

The study participants’ blood pressure (BP) was measured using a validated automated blood pressure monitor (Model M: 6, Omron Healthcare, Japan; 31). All participants were asked to relax for 5 min at room temperature before reporting their blood pressure results. Each person’s blood pressure was measured three times. The measurements were taken on the right arm using a monitor with a proper cuff size and short intervals between readings. For analysis, the average of the three measurements was recorded (32). Both systolic and diastolic blood pressure readings were reported for all the participants at three time points of the study.

### Blood sampling and analysis

Blood sampling was performed as reported previously (6). In brief, all blood samples at three different time points were collected in a way to ensure at least 8–10 h of fasting. In this way, pre-Ramadan (T1) and post-Ramadan (T3) fasting blood samples were collected in the morning, 7–8 a.m. before the morning breakfast. During Ramadan (T2), blood samples were collected at 11.00 a.m., approximately 8 h after the Sahar meal. Biochemical analyses were performed on blood samples following standard protocols at the Biology of Aging Laboratory, Singapore Immunology Network, Agency for Science Technology and Research, 8A Biomedical Grove, Singapore 138,648, Singapore. Consequently, blood lipid profile, blood glucose, hsCRP, and an extended panel of cytokines and chemokines were determined. Details of the procedures for the determination of these are provided in Supplementary File 2.

### Nutrition education strategy

The nutrition education strategy was partially based on adult learning theory (33). It consisted of four steps: (1) assessing the needs of the participants, (2) setting educational objectives, (3) choosing/using a variety of methods, and (4) assessing that learning occurred (33). The unique learning styles of participants were identified in a pilot study (visual, auditory, collage, etc.). Objectives that focus on what the participants will do with the contents of the nutrition education strategy in order to learn it were set, and the intervention was designed accordingly. Participants in the intervention group attended a multi-component nutrition education entitled “Dietary Education and Awareness for Ramadan: Healthy Eating for a Healthy Living during Ramadan” using three different modes of instruction (lecture/talk, leaflet, and picture collage). During the study period throughout the 4 weeks of Ramadan fasting, participants were kept in contact through mobile phones, personal contact, or in groups in Iftar Buffet.

### Procedure for nutrition education

The participants in the intervention group were gathered in three sessions for an average of 2 h. This was considered workable as previously prepared by Liu et al. (34). A mixture of Pashto and Urdu, as the local/national languages, was used during these instructions. All sessions were held in groups and were carried out by the investigator. The perception of the educational tools was pretested in a sample of 10 adults before the actual intervention.

**Figure 1:**
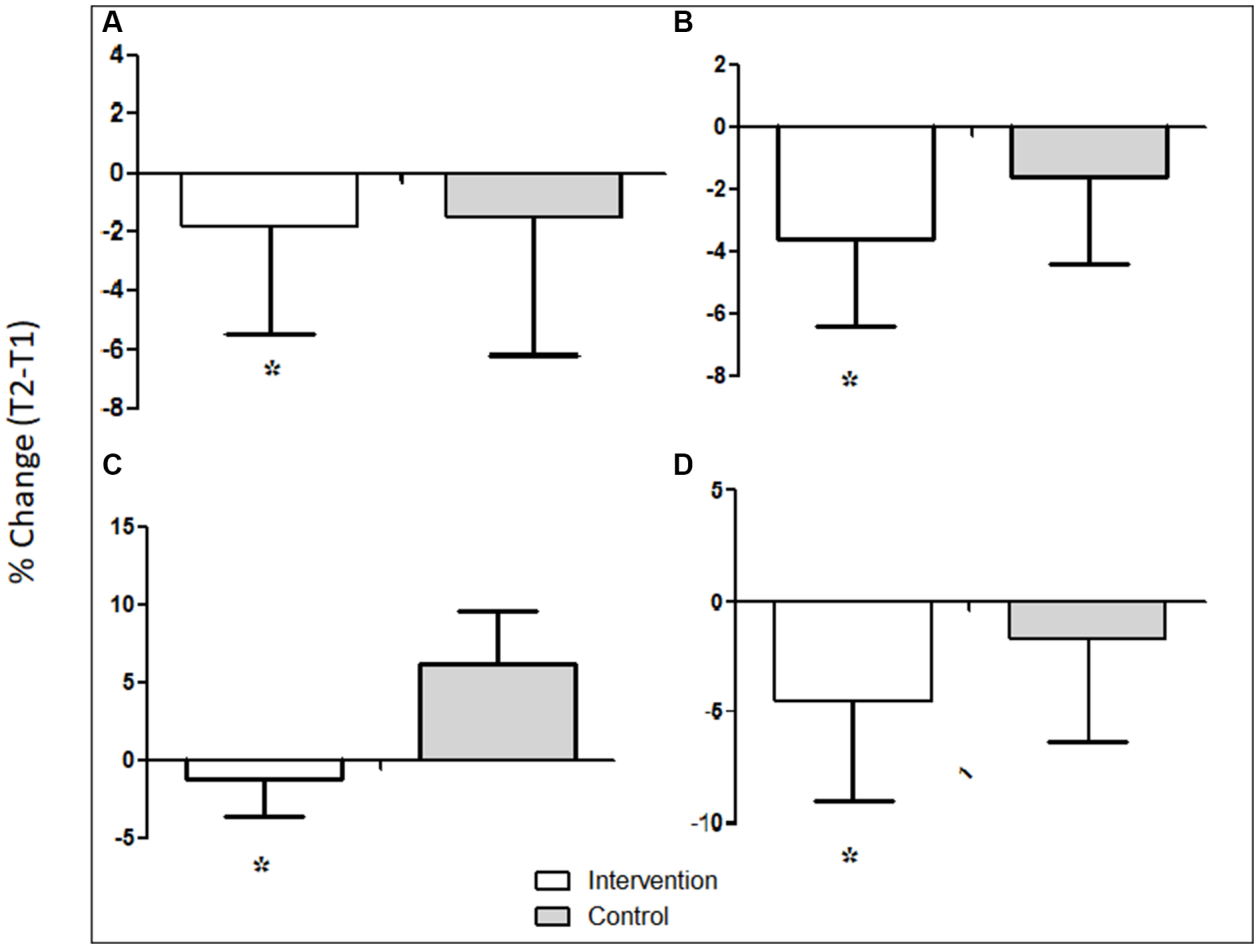
Mean percent reduction in anthropometrics. **(A)** Mean percent reduction in weight, **(B)** mean percent reduction in BMI, **(C)** mean percent reduction in WC, and **(D)** mean percent reduction in %BF. White bars represent the ‘intervention group’. Gray bar represent the ‘control group’.

The first session used a focal group technique. It was diagnostic in character. It was designed to identify limitations and/or barriers to healthy food consumption during Ramadan in the community. The second session was motivational in nature. It was formatted as a culinary workshop. The main purpose was to promote contact with different types of healthy foods for Ramadan fasting month, which therefore included the preparation and degustation of various recipes containing fruits and vegetables as the primary ingredients. This session also comprised a lecture guided by a poster and covered topics that addressed overall dietary quality, including: (a) identifying a preferable overall distribution of types of food in a diet using the plate model from the dietary guidelines for Pakistan (28) (Figure 1); (b) increasing the consumption of fresh fruits and vegetables, whole grains, and their products; (c) discouraging the consumption of fried foods (e.g., samosa, pakora, and kabab), foods high in fat and sugar (*jalaibee*, *sweets*, *puddings*, etc.), and (d) providing alternatives to low-nutrient snacks. This session also consisted of a brief summary of all topics discussed during the previous session and a picture collage was introduced where participants had to use pictures and place them in appropriate portions on a designed plate model. At the end of the second session, a leaflet consisting of topics discussed above together with a “sample menu” of iftar, dinner, sahar, and “alternatives to junky snacks (low nutrients snacks)” featuring mainly the local snack types. This was distributed to the participants as “take home lessons” to enhance their understanding of the whole session. The third and last session was essentially informative, mainly focusing on addressing the issues of nutritional recommendations for Ramadan, health benefits associated with fresh fruit and vegetable consumption in general, and during Ramadan in particular, ways to increase the consumption of such foods, and replacement of less healthy foods with fruit and vegetables. Question and answer sessions were conducted during the lecture to enable active participation.

In addition, participants in the intervention group with a normal baseline BMI (18.5–24.9) were encouraged to maintain their weight with no increase or decrease while still adjusting their diet to a healthier pattern and incorporating certain changes in their daily intake. The obese and overweight participants in the intervention group were encouraged to lose at least 5% of their weight at baseline by following a healthier pattern by incorporating certain changes in their daily intake. All participants in the intervention group were advised in a personalized manner. Targets in terms of weight and diet were defined and explained on an individual basis.

### Assessment of adherence to nutrition education

Adherence to nutrition education was assessed twice—1 day before the commencement of Ramadan fasting, just before the educational sessions, and a second time on the last day of Ramadan fasting. The purpose of assessing adherence to nutrition education at two time points was to know whether there was any difference in nutritional behavior between the two time points of the study (T1 and T2) in order to assess the overall impact of nutrition education on nutrition behavior and dietary intake.

The questionnaire used to assess nutrition education was adapted from the one developed by Permatasari et al. (35; Supplementary File 3). The questionnaire consisted of 13 questions, with answers graded using a Likert scale. Favorable questions (1–5) were scored as follows: 1 = strongly disagree, 2 = disagree, 3 = agree, and 4 = strongly agree. For unfavorable questions (6–13), this scoring was reversed as follows: 1 = strongly agree, 2 = agree, 3 = disagree, and 4 = strongly disagree. The questionnaire was completed before the intervention (baseline T1) and at the end of the intervention (i.e., at the end of Ramadan fasting, T2).

### Nutrition education impact assessment

It was necessary to assess how effective nutrition education was in modifying dietary behavior. This was done by estimating how much the participants in the intervention group demonstrated adherence to the Pakistan Dietary Guidelines for Better Nutrition (28). According to the PDGN, there must be 2–3 servings each of meat and pulses, vegetables, milk and milk products, fruits, and 4–5 times cereals and grains. The adherence to dietray advice given in the PDGN was calculated as reported previously (36). The ratings of the consumption of each food group (from 0 to 5 or the reverse) were adapted from Panagiotakos et al. (37) and are given in Table 1. The dietary adherence score included non-refined cereals and bread (whole bread, rice, pasta, and other grains), fruit, vegetables, legumes, fish, olive oil, meat and meat products, poultry, full-fat dairy products, sweets, and oils. For the intake of food items assumed to be close to the PDGN or higher (non-refined cereals, fruits, and vegetables), we allocated a score of 0 when the individual stated no consumption, a score of 1 when they stated the consumption of 1–4 servings/month, a score of 2 for 5–8 servings/month, a score of 3 for 9–12 servings/month, a score of 4 for 13–18 servings/month, and a score of 5 for more than 18 servings/month. Moreover, we included legumes, fish, and olive oil in this group after separating them from the meat and oil groups. In contrast, for the intake of food items assumed to be limited in PDGN (i.e., rare or monthly intake; meat and meat products, poultry, and full-fat dairy products), we allocated the scores on a reverse scale (i.e., 5, when individuals stated no intake, to 0, when they stated almost daily intake). Hence, the scores ranged from 0 to 60. Higher scores show better adherence to the Pakistan Dietary Guidelines for better nutrition.

**Table 1:** Scoring system for adherence to dietary PDGN score.

No.	Food groups	Frequency of consumption (Servings/Month)					
		Never	1–4	5–8	9–12	13–18	>18
1	Non-refined cereals and bread^a^	0	1	2	3	4	5
2	Fruit^b^	0	1	2	3	4	5
3	Vegetable	0	1	2	3	4	5
4	Legumes	0	1	2	3	4	5
5	Fish	0	1	2	3	4	5
6	Olive oil	0	1	2	3	4	5
7	Meat and meat products	5	4	3	2	1	0
8	Poultry	5	4	3	2	1	0
19	Full-fat dairy products	5	4	3	2	1	0
10	Sweets	5	4	3	2	1	0
11	Oils	5	4	3	2	1	0

a Whole-grain bread, rice, pasta etc.

b Fresh (e.g., apple, oranges, banana, grapes, etc.) and dried fruit, including dates.

### Ethical consideration

The ethics approval for the study was granted by the Human Research Ethics Committee of the Department of Human Nutrition, The University of Agriculture, Peshawar (Ref: HN-HREC/2017-022).

### Statistical analysis

Data obtained from the questionnaires were processed using Microsoft Excel and analyzed using SPSS v22 software. Participants’ baseline demographics were expressed as categorical data, with frequencies and percentages, or continuous data, with mean and standard deviation. Confidence intervals (CIs) were calculated and presented in separate tables in the supporting files (Supplementary File 4). Continuous data were analyzed using an independent sample t-test and categorical data by applying Fisher’s exact test. Adherence to PDGN was scored and displayed as continuous data. The differences in scores at the start and end of the study were measured using the Wilcoxon signed-rank test. Baseline differences between these variables were tested using the Mann–Whitney U-test. A value of p of <0.05 was considered significant.

## Results

Selected socio-demographic characteristics of the participants are presented in Table 2. The two groups were matched in age, employment status, education, monthly income, and nutritional status. The mean (SD) age of the case and control groups was 47.1 ± 6.7 years for the intervention group and 49.9 ± 7.5 years for the control group.

**Table 2:** Baseline demographics of the study participants.

Characteristics	Intervention group (*n* = 37)	Control group (*n* = 37)	*t*/*χ*^2^	*p*-value
Age [years; mean (SD)]	30.1 (12.2)	31.3 (13.5)	*t* = 0.65	0.511
Employment status, *n* (%)			*χ*^2^ = 1.412	0.265
Unemployed	20 (66.7)	27 (90.0)		
Employed	10 (33.3)	23 (76.7)		
Education, *n* (%)				
Primary school	12 (40)	18 (60.0)	*χ*^2^ = 4.52	0.386
Secondary school	10 (33.3)	12 (40.0)		
High School	4 (13.3)	4 (13.3)		
Undergraduate/Bachelor	4 (13.3)	4 (13.3)		
Monthly income^*^, *n* (%)			*χ*^2^ = 0.889	0.345
Below regional wage	16 (53.3)	18 (60.0)		
Above regional wage	14 (46.7)	12 (40.0)		
BMI, *n* (%)				
Normal Weight (18.5–24.9)	17 (56.7)	27 (90.0)	*χ*^2^ = 0.290	0.861
Overweight (25–30)	8 (26.7)	16 (53.3)		
Obese (>30)	5 (16.7)	7 (23.3)		

Results significant at *p* < 0.05; *χ*^2^ = Chi square test; BMI=Body mass index. *The Khyber Pakhtunkhwa Minimum Wages Act, 2013.

### Adherence to nutrition education

All participants in both the intervention group and control group completed the follow-up assessment at the two time points (T1 and T2) of the study. The mean start score for adherence to nutrition education was 16.0 (8.1; range: 9–24) for the control group and 16.6 (4.8; range: 9–27) for the intervention group (*p* = 0.406). The end scores were 17.1 (9.9; range: 10–25) and 42.5 (13.2; range: 30–48) in the control and intervention groups, respectively. There was a significant improvement in the intervention group’s adherence to nutrition education test scores, with an absolute increase in a mean score of 25.6 (*p* < 0.0001). No significant difference was observed in the control group test scores (absolute difference 1.3; *p* = 0.224).

## Effect of nutrition education

### Primary outcome

#### Dietary adherence to the PDGN score

Table 3 shows mean (SD) dietary adherence scores to PDGN at three time points for intervention and control groups. In general, the mean (SD) dietary adherence to PDGN scores at T2 for all parameters for the intervention group was significantly higher as compared to T1. These scores remained stable to some extent even after the 4-week post-Ramadan period (T3). The total dietary adherence to PDGN score at T2 was significantly higher as compared to T1 for the intervention group (36.7 vs. 14.2; *p* < 0.05). The total dietary adherence to the PDGN score for the control group between T1 and T2 differed non-significantly (14.0 vs. 11.8; *p* > 0.05).

**Table 3:** Mean (SD) adherence to dietary PDGN scores.

	Intervention^1^ (*n* = 37)			Control^1^ (*n* = 37)			*p*-value^*^
	T1	T2	T3	T1	T2	T3	
Non-refined cereals and bread	1.2 (1.1)^a^	3.1 (1.4)^b^	1.5 (1.1)^a^	1.3 (0.9)^a^	1.3 (1.2)^a^	1.4 (1.1)^a^	<0.0001
Fruit	1.9 (1.7)^a^	4.4 (2.1)^b^	2.5 (1.2)^b^	1.8 (0.1)^a^	1.9 (1.1)^a^	1.5 (1.2)^a^	<0.0001
Vegetable	3.9 (2.1)^a^	4.5 (2.7)^b^	4.5 (1.5)^a^	3.9 (2.1)^a^	2.9 (1.1)^b^	3.7 (1.1)^a^	0.05
Legumes	1.6 (1.2)^a^	3.6 (2.3)^b^	2.6 (2.1)^b^	1.6 (1.2)^a^	1.8 (1.2)^a^	1.7 (1.2)^a^	<0.0001
Fish	1.8 (1.3)^a^	3.6 (2.5)^b^	2.7 (1.3)^b^	1.6 (1.2)^a^	1.3 (1.1)^a^	1.5 (0.4)^a^	<0.0001
Vegetable/Olive oil	0.7 (1.2)^a^	2.1 (1.2)^b^	1.2 (1.5)^b^	0.8 (0.4)^a^	0.9 (0.4)^a^	0.2 (0.1)^b^	0.01
Red Meat and meat products	0.5 (1.1)^a^	1.4 (1.1)^b^	0.5 (0.2)^a^	0.4 (0.1)^a^	0.5 (0.4)^a^	0.5 (0.2)^a^	<0.0001
Poultry	0.5 (1.0)^a^	2.6 (1.3)^b^	1.2 (0.6)^b^	0.5 (0.2)^a^	0.8 (0.4)^a^	0.6 (0.3)^a^	<0.0001
Full-fat dairy products	1.1 (1.1)^a^	3.6 (2.1)^b^	1.1 (0.1) ^a^	1.3 (0.4)^a^	1.2 (1.2)^a^	1.2 (0.4)^a^	<0.0001
Sweets	0.5 (0.4)^a^	4.1 (2.7)^b^	1.4 (0.5) ^b^	0.2 (0.1)^a^	0.7 (0.2)^a^	0.5 (0.4)^a^	<0.0001
Ghee	0.5 (0.3)^a^	3.7 (2.1)^b^	1.7 (1.1)^b^	0.8 (0.4)^a^	0.7 (0.2)^a^	0.5 (0.4)^a^	<0.0001
Total adherence to PDGN	14.2 (9.2)^a^	36.7 (12.7)^b^	20.9 (11.9)^b^	14.2 (6.3)^a^	14 (6.3)^a^	11.8 (5.9)^a^	<0.0001

* *p*-value, based on mean differences between T1 and T2 for intervention and control groups.

1 Means followed by the same letter do not differ significantly. T1 = baseline; T2 = at the end of Ramadan fasting month; T3 = 1 month post-Ramadan; Two Way ANOVA, with *p* < 0.05.

## Secondary outcomes

### Changes in anthropometrics

As shown in Table 4, at baseline, the intervention and control groups had the same anthropometrics and body composition parameters (p, for all trends>0.05). Details of the ANOVA associated with Table 4 are given in Supplementary File 4. Overall, there was a general trend of a decrease in body weight, BMI, WC, and % BF at T2 and then a slight increase by T3. However, most of these variables were still lower at T3, despite an increase after T2 during the post-Ramadan period. The time x group interactions for BMI, WC, and % BF were as follows: significant for BMI [*F* (2, 116) =10; *p* < 0.0001], WC [*F*_(2, 115)_ = 10.5; *p* < 0.0001], and non-significant for % BF [*F*(2, 116) = 1.5; *p* = 0.221].

**Table 4:** Mean (SD) of Anthropometrics of Intervention and Control Groups.

Variables	Intervention (*n* = 30)			Control (*n* = 30)			Two-way analysis of variance (*p*-value)^*^		
	T1	T2	T3	T1	T2	T3	Group	Time	Group × Time
Weight	74.2 (10.8)^a^	72.9 (10.68)^b^	73.8 (10.73)^a^	71.5 (10.54)^a^	70.4 (10.39)^a^	71.7 (10.59)^a^	0.925	<0.0001	<0.0001
BMI	25.1 (3.95)^a^	24.6 (3.8)^b^	24.9 (3.9)^c^	24.5 (3.62)^a^	24.1 (3.60)^a^	24.5 (3.60)^a^	0.901	<0.0001	<0.0001
WC	90.0 (10.8)^a^	88.9 (10.49)^b^	89.9 (10.47)^a^	90.0 (8.9)^a^	95.6 (8.77)^a^	88.2 (8.93)^a^	0.116	<0.0001	<0.0001
%BF	29.1 (6.68)^a^	24.8 (5.12)^b^	27.2 (5.84)^c^	31.1 (6.81)^a^	29.4 (5.88)^a^	29.0 (6.08)^c^	0.294	<0.0001	0.217

^1^Means followed by the same letter do not differ significantly. One-way ANOVA, with *p* < 0.05; T1 = baseline; T2 = at the end of Ramadan fasting month; T3 = 1 month post-Ramadan; Two Way ANOVA, with *p* < 0.05.

* *p*-value, Two Way ANOVA, *p* < 0.05.

Figure 1 shows that compared to the control group, participants in the intervention group had a greater percent reduction in weight (−1.8 vs. −1.5%.), BMI (−3.6 vs. −1.6%), WC (−1.2 vs. 6.2%), and % BF (−4.4 vs. −1.1%; *p*, for all trends < 0.05).

Table 5 shows the mean (SD) intake of energy, macronutrients, and fiber. In general, mean energy intake significantly differed at three time points of the study in both the intervention and control groups. Details of these analyses associated with Table 5 are given in Supplementary File 4.

**Table 5:** Mean (SD) of nutrients intake of Intervention and Control Groups.

Variables	Intervention (*n* = 30)			Control (*n* = 30)			*p*-value^*^		
	T1	T2	T3	T1	T2	T3	Group	Time	Group × Time
Energy (Kcal)	2052	2,191	2,322	2,302	2,193	2,389	<0.0001	0.0003	0.0012
Carbohydrates (g)	301.9	332.5	347.9	353.2	345.3		<0.0001	<0.0001	<0.0001
Total Protein (g)	71.2	70.4	72.1	71.3	72.5	72.3	0.542	0.636	0.856
Fats (g)	62.2	64.4	71.3	67.1	58.0	71.9	0.854	<0.0001	0.021
Fiber (g)	11.1	10.7	10.5	10.9	1.0	10.2	0.654	0.340	0.759

^1^Means followed by the same letter do not differ significantly. One-way ANOVA, with *p* < 0.05; T1 = baseline; T2 = at the end of Ramadan fasting month; T3 = 1 month post-Ramadan; Two Way ANOVA, with *p* < 0.05.

* *p*-value, Two Way ANOVA, *p* < 0.05.

**Figure 2:**
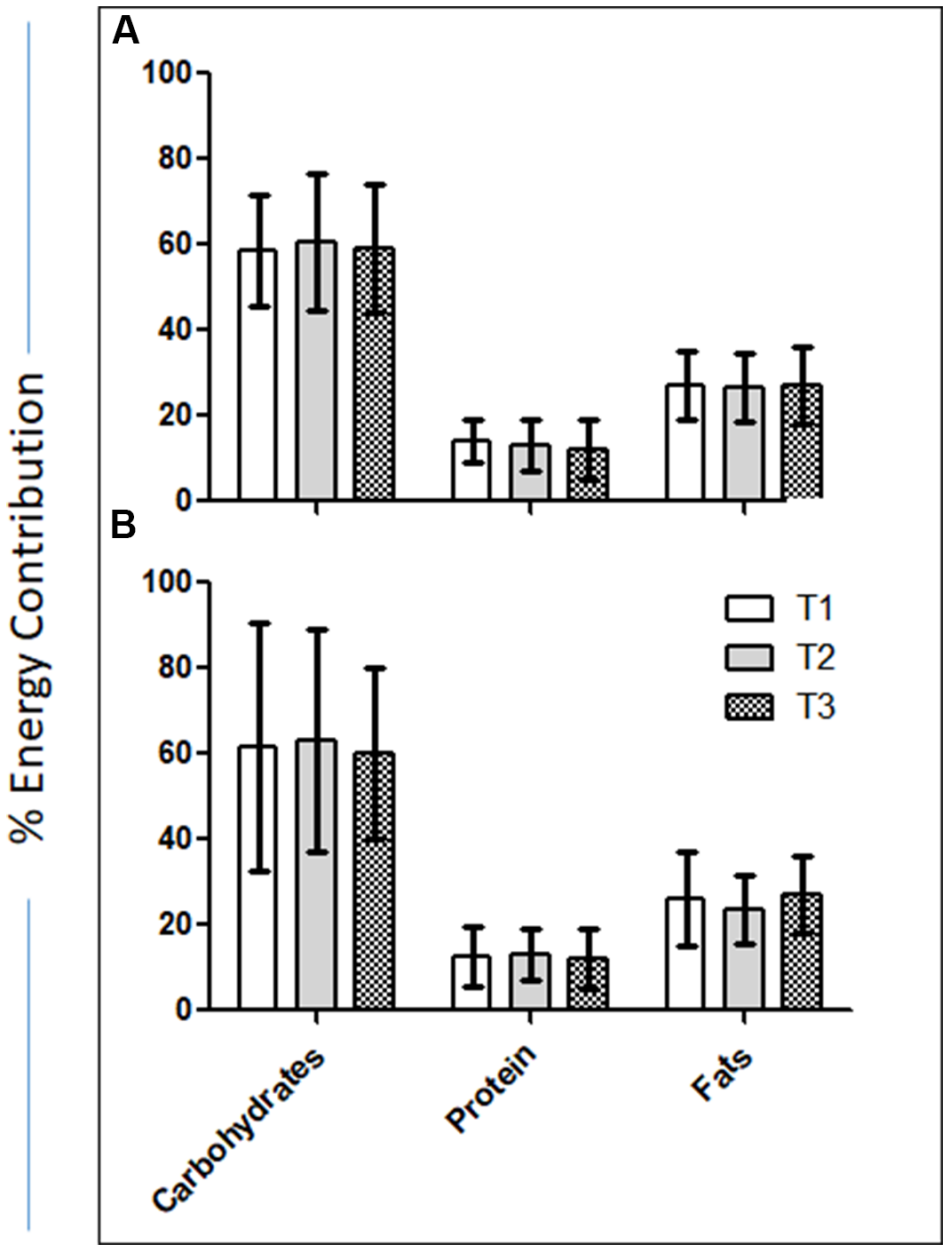
**(A)** Percent change in the intake of saturated and unsaturated fats between T1 and T2. **(B)** Percentage of energy distribution in carbohydrates, protein, and fats in the intervention and control groups.

Figure 2A shows the %age of energy distribution in carbohydrates, protein, and fats in the intervention and control groups. The percent energy contribution by carbohydrates in the intervention and control groups slightly increased at T2 compared to T1 and then decreased at T3. The percent energy contribution by protein slightly decreased at T2 as compared to T1 in the intervention group but increased in the control group. The percent energy contribution by fats also slightly decreased at T2 as compared to T1 in the intervention and control groups.

Figure 2B shows the %age change in the intake of saturated and unsaturated fats between T1 and T2. In the intervention group, saturated fat intake decreased by 20.9%, while unsaturated fat intake increased by 147.8%. In the control group, the saturated fat intake decreased by 8.2%, and the intake of unsaturated fats decreased by 78.4%.

### Changes in biochemical profiles

Table 6 shows the mean (SD) of various blood biochemicals at three time points of the study. Compared to T1, mean (SD) values of blood glucose, cholesterol, sodium, CRP, and diastolic and systolic blood pressure were lower at T2 for the intervention group. These differences were less evident for the control group. Details of the ANOVA associated with Table 6 are given in Supplementary File 4.

**Table 6:** Mean (SD) of selected bio-chemicals and diastolic and systolic blood pressure.

Variables	Intervention (*n* = 30)			Control (*n* = 30)			Two-way analysis of variance (*p*-value)		
	T1	T2	T3	T1	T2	T3	Group	Time	Group × Time
Glucose (mg/dl)	123.4 (14.93)^a^	98.65 (14.7)^b^	105 (13.2)^c^	120.9 (21.4)^a^	101.3 (13.59)^b^	100.03 (27.85)^c^	0.698	<0.0001	0.397
Cholesterol (mg/dl)	149.5 (65.3)^a^	84.48 (74.2)^b^	150.1 (112.4)^a^	133.5 (82.1)^a^	107.9 (76.12)^a^	96.3 (78.81)^c^	0.340	0.0503	0.0330
HDL (mg/dl)	35.9 (9.11)^a^	34.4 (12.22)^a^	33.4 (11.88)^c^	30.9 (9.2)^a^	32.4 (8.4)^a^	30.4 (11.2)^a^	<0.0001	<0.0001	<0.0001
LDL (mg/dl)	112.6 (74.2)^a^	50.1 (32.21)^b^	116.7 (57.31)^a^	85.6 (23.11)^a^	75.5 (34.34)^b^	76.9 (31.11)^c^	<0.0001	<0.0001	<0.0001
Sodium (mg)	3.2 (0.32)^a^	3.0 (0.56)^a^	3.2 (0.81)^a^	2.9 (0.21)^a^	2.7 (0.24)^a^	3.0 (0.33)^a^	<0.0001	<0.0001	<0.0001
CRP (pg/ml)	3.4 (1.11)^a^	2.9 (1.25)^b^	5.47 (2.11)^c^	4.1 (2.08)^a^	3.6 (1.80)^b^	3.3 (2.18)^c^	<0.0001	0.601	0.521
Diastolic BP (mmHg)	80.5 (1.52)^a^	78.3 (2.39)^a^	79.8 (4.11)^a^	80.8 (2.12)^a^	79.2 (2.11)^a^	80.4 (1.37)^a^	<0.0001	<0.0001	<0.0001
Systolic BP (mmHg)	123 (7.51)^a^	116.2 (6.11)^b^	112.0 (6.10)^c^	123.2 (7.40)^a^	114.8 (6.35)^b^	122.6 (4.77)^a^	<0.0001	<0.0001	<0.0001

^*^p-value, Two Way ANOVA, *p* < 0.05. ^1^Means followed by the same letter do not differ significantly. One-way ANOVA, with *p* < 0.05.

### Changes in cytokines/chemokines levels

Table 7 shows the mean (SD) concentrations of cytokines/chemokines stratified by the intervention and control groups and by the three time points of the study. The table also gives the results of the ANOVA. For the intervention group, mean (SD) values of pro-inflammatory cytokines/chemokines (IL-2, IL-7, TNF-α, and Resistin) were higher at T2 as compared to T1 (*p*, for all trends<0.05). For these cytokines/chemokines, the control group followed almost the same trend. In case of pro-inflammatory cytokines, for the intervention group, the levels of these cytokines at T2 were also higher as compared to T1. For the control group, with the exception of IL-1 and TGF-α, the mean (SD) values of these cytokines were lower at T2 as compared to T1.

**Table 7:** Mean (SD) of cytokines/chemokines.

Variables	Intervention (*n* = 30)			Control (*n* = 30)			*p*-value^*^		
	T1	T2	T3	T1	T2	T3	Group	Time	Group × Time
IL-2 (pg/ml)	1.37 (0.50)^a^	1.99 (1.05)^b^	1.65 (0.79)^c^	1.6 (0.98)^a^	1.84 (1.09)^a^	1.62 (0.86)^a^	0.393	<0.0001	0.497
IL-7 (pg/ml)	5.2 (4.3)^a^	5.4 (2.1)^a^	5.8 (2.1)^a^	5.1 (4.2)^a^	4.9 (2.3)^a^	5.0 (5.2)^a^	0.299	0.853	0.722
TNF-α (pg/ml)	36.45 (32.42)^a^	75.23 (61.18)^b^	52.1 (41.63)^c^	44.1 (3.7)^a^	56.1 (17.1)^b^	54.9 (13.01)^c^	0.792	0.008	0.219
Resistin (pg/ml)	3,655 (1777)^a^	3,976 (3083)^b^	3,786 (5446)^c^	3,655 (2015)^a^	3,986 (1482)^b^	3,716 (1633)^a^	<0.0001	0.662	0.700
IL-1 RA (pg/ml)	81.0 (87.01)^a^	140.4 (127.2)^b^	92.14 (68.25)^a^	111 (97.69)^a^	117.1 (77.61)^b^	126 (68.26)^c^	0.383	0.077	0.0871
IL-4 (pg/ml)	46.4 (28.52)^a^	54.33 (33.74)^b^	52.64 (34.36)^c^	44.3 (32.37)^a^	56.1 (29.89)^b^	54.9 (31.91)^b^	0.744	0.771	0.281
IL-17 A (pg/ml)	5.2 (3.15)^a^	5.45 (3.39)^a^	5.89 (3.29)^c^	5.32 (2.94)^a^	4.65 (4.02)^b^	5.83 (2.47)^a^	<0.0001	0.667	0.553
sCD40 (pg/ml)	20.47 (8.01)^a^	20.35 (10.19)^a^	13.51 (11.78)^c^	44.05 (8.7)^a^	56.02 (11.4)^b^	54.61 (9.56)^b^	0.001	0.622	0.392
TGF-α (pg/ml)	6.82 (3.3)^a^	7.91 (5.52)^a^	7.83 (3.98)^a^	7.08 (4.78)^a^	6.84 (3.05)^a^	6.93 (3.69)^a^	<0.0001	0.621	0.504

^1^Means followed by the same letter do not differ significantly. One-way ANOVA, with *p* < 0.05.

* *p*-value, Two Way ANOVA, *p* < 0.05.

**Figure 3:**
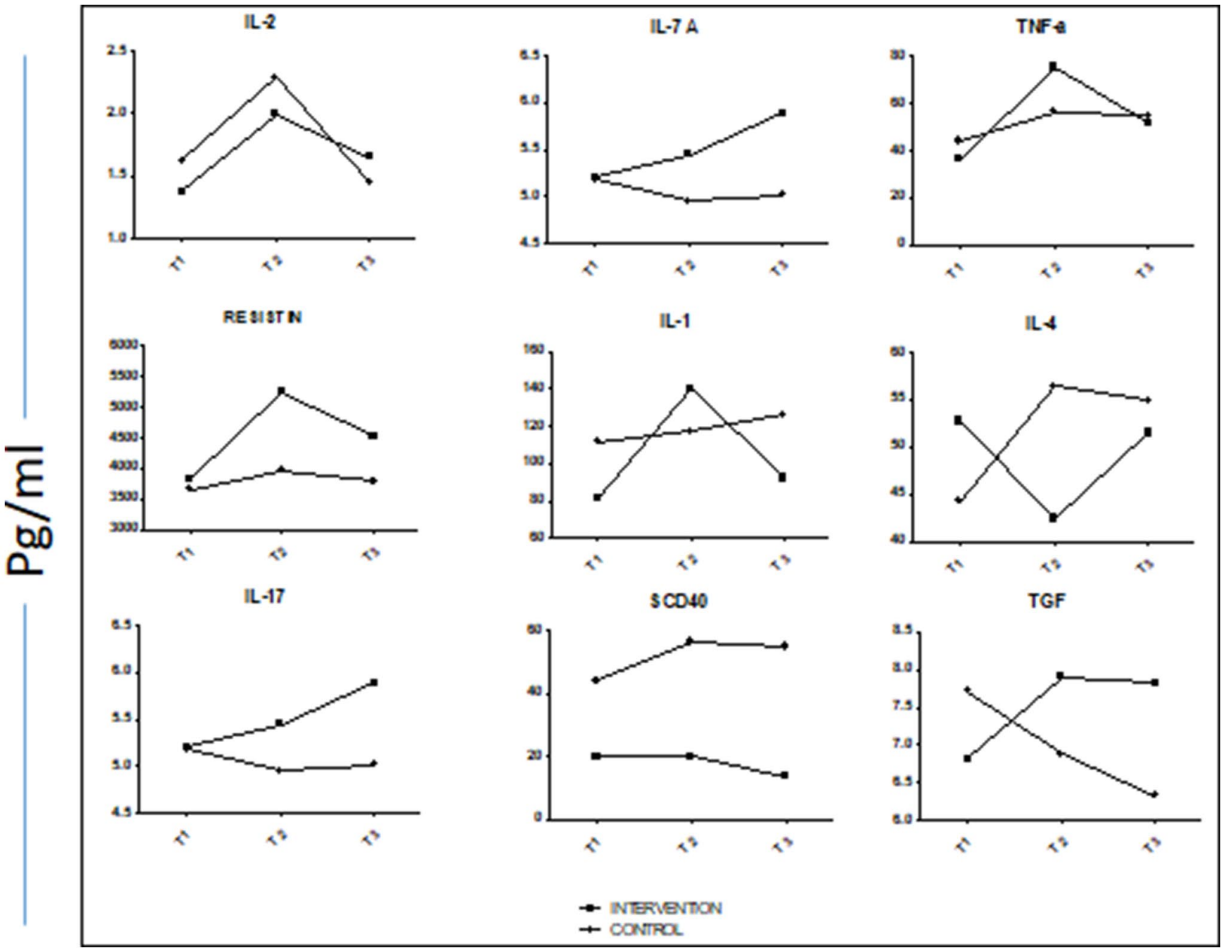
Trend lines of mean concentrations of selected cytokines/chemokines at the three time points both of control and intervention groups.

Changes in the cytokines/chemokines levels through the three time points of the study for both the intervention and control groups are also depicted in Figure 3. As evident, in most of the cases, the blood concentration of cytokines/chemokines elevated during Ramadan fasting and then either further went up or came back closer to the baseline level at the post-Ramadan period.

## Discussion

### Major findings

1. Assessment of nutrition education showed a significant improvement in the intervention group adherence test scores, while no significant difference was observed in the control group test scores. This shows that nutrition education may be a successful strategy in bringing positive and healthy dietary changes in Ramadan fasting.
2. Nutrition education was successful in improving dietary adherence to PDGN scores in the intervention group. The total dietary adherence to the PDGN score significantly improved at T2 compared to T1 for the intervention group (mean scores: 36.7 vs. 14.2; *p* < 0.05). The total dietary adherence to PDGN score for the control group between T1 and T2 differed non-significantly (14.0 vs. 11.8; *p* > 0.05).
3. Compared to control, there was significant weight loss in the intervention group at T2 (*p* > 0.05).
4. Overall, there was a general trend of a decrease in body weight, BMI, WC, and % BF at T2 and then a slight increase by T3. However, most of these variables were still lower at T3 despite an increase after T2 during the post-Ramadan period.
5. Compared to the control group, participants in the intervention group had a greater percent reduction in weight (−1.8 vs. −1.5%.), BMI (−3.6 vs. −1.6%), WC (−1.2 vs. 6.2%), and % BF (−4.4 vs. −1.1%).
6. Compared to T1, mean (SD) values of blood glucose, cholesterol, sodium, CRP, and diastolic and systolic blood pressure were lower at T2 for the intervention group. These differences were less evident for the control group.
7. Concentration of most of the cytokines/chemokines elevated during Ramadan fasting and then either further went up or came back closer to the baseline level at the post-Ramadan period in both the intervention and control groups.
8. In the intervention group, saturated fat intake decreased by 20.9%, while unsaturated fat intake increased by 147.8%. In the control group, the saturated fat intake decreased by 8.2% and the intake of unsaturated fats decreased by 78.4%.

**Figure 4:**
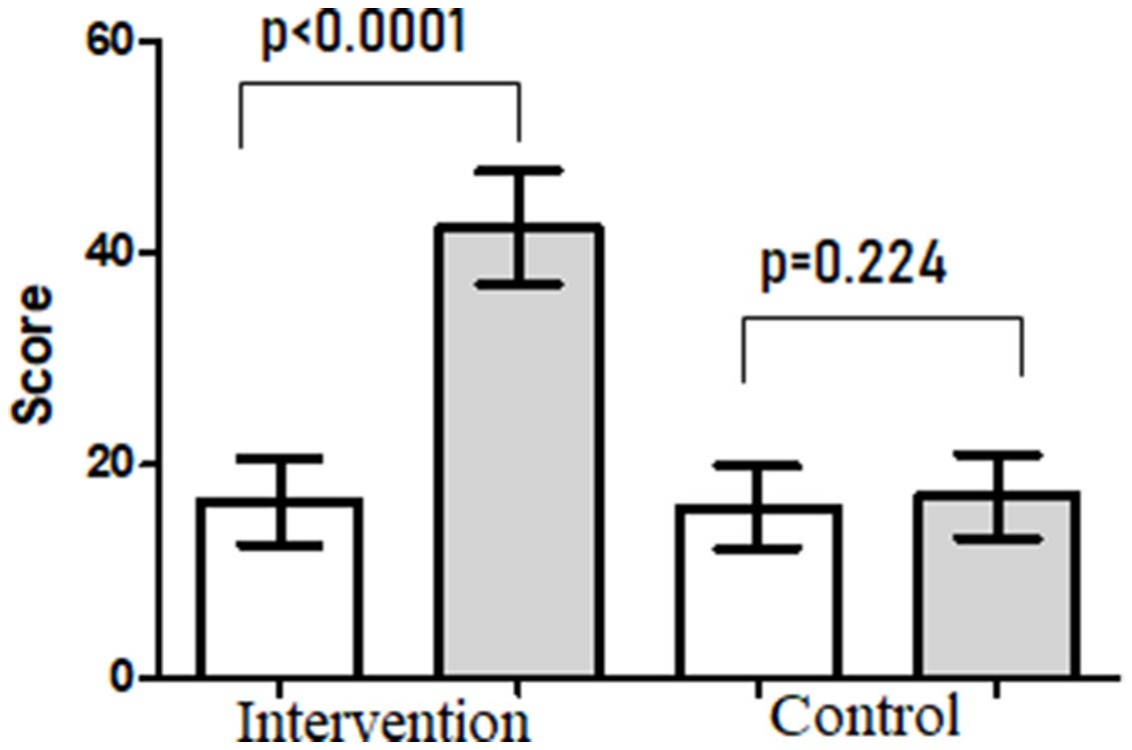
Dietary adherence at the start and end of the study.

Ramadan fasting brings numerous changes in dietary intake. These changes may have beneficial or adverse impacts on overall health, as fasting for 29–30 days is a relatively longer period. Ramadan is now of a ‘feasting’ than ‘fasting’ occasion. This may modify dietary patterns in an unhealthy way. The likelihood of unhealthy dietary practices in Ramadan is higher. Previous studies have recommended a dire need for nutrition education for Ramadan fasting. As part of this study, we investigated the effect of formal nutrition education before the start of Ramadan on dietary adherence to the PDGN score (as our primary outcome), weight, nutritional intake, and selected biochemicals and cytokines (as our secondary outcomes) in a group of 30 adult male participants compared to weight and BMI-matched 30 participants in the control group. Adherence rate and adherence to nutrition education were very high (Table 3), and hence the non-adherence rate was very, i.e., 18.9% (7 out of 37), which was very low compared to 50 and 80% of non-adherence rate for chronic disease management, including medication and lifestyle changes (38). Nutrition education improved adherence to dietary advice as there was a significant improvement in the intervention group test scores, with an absolute increase in the mean score of 25.6, while the control group, who received no nutrition education, failed to improve their adherence to PDGN score (Figure 4). The concept of ‘adherence’ recognizes the patient’s right to choose whether or not to follow advice and implies an individual’s active participation in the treatment regimen [Cohen (39)]. Thus, poor adherence can be a serious threat to the overall health and wellbeing of an individual (40) and also carry an economic burden (41). Adherence is particularly important in the context of chronic diseases that require long-term therapy and a number of permanent rather than temporary changes in lifestyle behaviors, including diet (38). The extent to which risk-reduction interventions proved to be as effective in research settings as in individuals’ real-life settings depends on the individual’s adherence to treatment advice. In that regard, results from a randomized clinical trial that wanted to assess the adherence to and effectiveness of various types of diets revealed that the level of adherence to dietary advice, rather than the type of diet, was the key determinant of effective weight loss (42).

As a primary outcome of interest, dietary adherence to the PDGN score improved in the intervention group, who received nutrition education, as compared to the control group, who did not get any nutrition education but followed their usual dietary intake patterns throughout the Ramadan fasting month. Dietary adherence to PDGN scores for all parameters in the intervention group significantly improved after they received nutrition education. The total dietary adherence to the PDGN score assessed at the end of Ramadan fasting was significantly higher as compared to the baseline score (36.7 vs. 14.2; *p* < 0.05). On the other hand, the total dietary adherence to PDGN score for the control group at the end of Ramadan did not differ much from the dietary adherence to PDGN score at baseline (14.0 vs. 11.8; *p* > 0.05; Table 2). These data from the present study show that nutrition education at the start of Ramadan brings positive and healthy changes in the dietary patterns of adult fasting individuals. To the best of our knowledge, there has been no study conducted on otherwise normal adult subjects investigating the impact of nutrition education on dietary modification, and we are therefore unable to compare our results with previous studies during Ramadan fasting. Nevertheless, nutrition education studies done on diabetic patients have shown the overall efficacy of such strategies (43). Participation in structured education programs has shown improvement in their body weight, BMI, and dietary fiber intake in addition to self-empowerment, knowledge of diabetes, and self-management skills (44, 45). In a systematic review of randomized controlled trials of the effectiveness of self-management training in people with type 2 diabetes, educational interventions that involved patient collaboration were more effective than didactic interventions in improving short-term glycemic control (< 6 months), weight, and lipid profiles (46).

As a secondary outcome, the present study found significant mean weight loss in the intervention group who received nutrition education. The loss was much more pronounced compared to that in the control group, which might indicate an effect of nutrition education on healthy weight management. Other anthropometric variables of interest (e.g., BMI, WC, and %BF) also changed during Ramadan fasting as expected. Participants in the intervention group had a greater percent reduction in weight (−1.8 vs. −1.5%.), BMI (−3.6 vs. −1.6%), WC (−1.2 vs. 6.2%), and % BF (−4.4 vs. −1.1%; Figure 1). In addition, the results of the present study also showed improvements in the quality of fat intake. In the intervention group, saturated fat intake decreased by 20.9%, while unsaturated fat intake increased by 147.8%. In the control group, the saturated fat intake decreased by 8.2% and the intake of unsaturated fats decreased by 78.4% (Figure 2B). These positive changes in the nutrient intake, particularly the quality of fat intake, in the intervention group, might have been reflected in a much-pronounced improvement in blood, glucose, cholesterol, sodium, CRP, and diastolic and systolic blood pressure in the intervention groups compared to the control group (Table 6). One of the reasons for these positive effects may be that the participants in the intervention group with improvements in their dietary adherence to the PDGN score had a much more balanced diet with increased servings of grains, vegetables, and fruits and a lower amount of red meat, dairy products, and hydrogenated cooking oil (ghee). Recently, much emphasis has been placed on certain dietary patterns to prevent chronic diseases, for example, the Mediterranean diet, the healthy American diet, and the vegetarian diet (47). All of these dietary patterns incorporate traditional advice to eat more fresh fruits, vegetables, and whole-grain cereals. Therapeutic diets such as the Dietary Approaches to Stop Hypertension (DASH) (48) and the dietary portfolio recommended in the Canadian Cardiovascular Society guidelines (49) to lower cholesterol also emphasize these principles and consistently result in large reductions in blood pressure and lipids when taken under metabolically controlled conditions (50–52). However, despite many efforts to encourage the general public to increase plant food consumption, the response has been slow (48). In this context, the results of the present study show that Ramadan fasting combined with nutrition education has pronounced positive effects on dietary behavior.

## Conclusion

Nutrition education before Ramadan fasting is an effective strategy to modify dietary behavior in such a way that it improves adherence to dietary guidelines, hence affecting weight management goals.

## Limitations

Measurement of adherence to prescribed dietary advice typically involves the following: (1) the assessment of what the client eats through self-reported methods (e.g., 24-h recall, food records, food frequency questionnaires, and diet history) and (2) the determination of the degree to which the diet approximates the recommended dietary plan (e.g., the difference between clients’ recommended macronutrient goals and their self-reported intake). Although sparsely used, more objective measures of adherence to diets also exist (e.g., 24-h urinary sodium excretion to assess adherence to a low sodium diet) (53). However, there is no gold standard for the accurate determination of dietary intake. Self-report of energy intake is a characteristic inherent to nutrition-related topics and is found to be underestimated compared to objective measures such as resting energy expenditure assessed by indirect calorimetry (54). Underreporting energy intake is observed more frequently in female participants as compared to male participants (55), in older individuals as compared to young people (56), and in obese individuals as compared to normal-weight individuals (57). Although self-reported measures are often regarded as more susceptible to bias (e.g., over-reliance on memory; daily dietary variability; report error related to portion size and meal composition; and social desirability), they are a direct, simple, and inexpensive method and are readily available for use in practice. Self-reported measures can be improved and validated by using multiple measures of adherence and controlling statistically for bias or by using constructs, for example, body weight, blood pressure, or plasma cholesterol concentrations (58–60).

## Data availability statement

The raw data supporting the conclusions of this article will be made available by the authors, without undue reservation.

## Ethics statement

The studies involving human participants were reviewed and approved by the ethical approval for the study was granted by the Human Research Ethics Committee of the Department of Human Nutrition, The University of Agriculture, Peshawar (Ref: HN-HREC/2017-022). The patients/participants provided their written informed consent to participate in this study.

## Author contributions

RG, IK and IA have substantial contribution to the concept and design of the study, data collection, analysis and interpretation. IH and MH assisted in data collection and data entry. RG, IK, AMA, and IA drafted the article and revised it critically for important intellectual content. NS, HC, SS and AA helped in data entry, data interpretation, reviewing the revised draft. All authors contributed to the final revision of the article and all approved the final submitted version.
