# Supplementary material for: Ramadan-specific nutrition education improves cardio-metabolic health and inflammation—a prospective nutrition intervention study from Pakistan
Source: Front Nutr. 2023 Dec 22;10:1204883. doi: 10.3389/fnut.2023.1204883 (PMC10798056; doi:10.3389/fnut.2023.1204883)
Supplement: Supplementary file 1 [file Data_Sheet_1.PDF]

Annexure-1

Dietary Education and Awareness for Ramadan (DEAR)

1. A healthy diet includes a daily consumption of 4-6 servings of the following foods: bread; grains, pasta, rice and potatoes. A serving of pasta or rice is generally a plate, a serving of grains is generally a bowl, a serving of pan is generally 3 or 4 slices or a role (40-60 grams) and a serving of potatoes is generally 1 large or 2 small potatoes (150-200 gram).
2. A healthy diet includes the daily consumption of 3 or more servings of fresh fruits. An example of a serving would be a medium piece, a cup of cherries, two slices of melon (A serving equals 120-200 gram).
3. A healthy diet includes a daily consumption of 2 or more serving of vegetables, raw or cooked without fat. A serving of these foods would be, for example: a plate of salad, a plate of cooked vegetables, 1 large tomato, 2 carrots (A serving equals 150-200 gram).
4. A healthy diet includes the daily consumption of 2 to 4 servings of milk and dairy products. A serving is considered to be, a glass of milk (200-250 ml), 2 yogurts (200-250 gram) or 3 slices of cheese (40-60 gram of cured chees or 80-125 gram of fresh cheese).
5. A healthy diet includes weekly consumption of 3 to 4 serving of fish, or the equivalent of one individual fillet (125-150 grams).
6. A healthy diet includes the weekly consumption of 3 to 4 servings of meat low in fat, with no visible fat, and with no skin on fowl. A serving is a small fillet, or a quarter chicken or rabbit (125-150 gram).
7. A healthy diet consists of a weekly consumption of 2 to 4 serving of legumes, (chickpeas, lentils….) or in other words, the equivalent to a plate of legumes (60-80 grams raw)
8. A healthy diet consists of a weekly consumption of 3 to 7 servings or nuts. One serving equals a handful (20-30 grams).
9. A healthy diet consists of a daily consumption of 1.5 to 2 liters of water.

Annexure-II

Questionnaire: Adherence to Nutrition Education

	Agree	Strongly Agree	Disagree	Strongly Disagree
I eat to eat according to the schedule recommended by the Dietitian.				
I eat types/amount of foods according to the recommendation from the Dietitian.				
I consume/would like to consume food that contains more vitamins and minerals.				
I eat food that is rich in protein such as meat, eggs or soybean.				
I eat fruits and vegetables every day as recommended by the Dietitian.				
I weigh myself every day.				
I do not want to follow the dietary recommendations from Dietitian because it is inconvenience for me.				
I have been too busy with my work and I do not eat according to the recommended meal time.				
I often consume sweet or sugary foods and drinks				
I often eat fast food or fatty food (such as fries or animal innards).				
I eat more than three major meals every day				
I often consume salty food, such as dried fish				
I often have snacks outside regular meal times				
