# Supplementary material for: Ramadan-specific nutrition education improves cardio-metabolic health and inflammation—a prospective nutrition intervention study from Pakistan
Source: Front Nutr. 2023 Dec 22;10:1204883. doi: 10.3389/fnut.2023.1204883 (PMC10798056; doi:10.3389/fnut.2023.1204883)
Supplement: Supplementary file 2 [file Data_Sheet_2.docx]

**SUPPLEMENTARY FILE 1 (S1)**

**DIETARY EDUCATION AND AWARENESS FOR RAMADAN (DEAR)**

1. A healthy diet includes a daily consumption of 4-6 servings of the following foods: bread; grains, pasta, rice and potatoes. A serving of pasta or rice is generally a plate, a serving of grains is generally a bowl, a serving of pan is generally 3 or 4 slices or a role (40-60 grams) and a serving of potatoes is generally 1 large or 2 small potatoes (150-200 gram).
2. A healthy diet includes the daily consumption of 3 or more servings of fresh fruits. An example of a serving would be a medium piece, a cup of cherries, two slices of melon (A serving equals 120-200 gram).
3. A healthy diet includes a daily consumption of 2 or more serving of vegetables, raw or cooked without fat. A serving of these foods would be, for example: a plate of salad, a plate of cooked vegetables, 1 large tomato, 2 carrots (A serving equals 150-200 gram).
4. A healthy diet includes the daily consumption of 2 to 4 servings of milk and dairy products. A serving is considered to be, a glass of milk (200-250 ml), 2 yogurts (200-250 gram) or 3 slices of cheese (40-60 gram of cured chees or 80-125 gram of fresh cheese).
5. A healthy diet includes weekly consumption of 3 to 4 serving of fish, or the equivalent of one individual fillet (125-150 grams).
6. A healthy diet includes the weekly consumption of 3 to 4 servings of meat low in fat, with no visible fat, and with no skin on fowl. A serving is a small fillet, or a quarter chicken or rabbit (125-150 gram).
7. A healthy diet consists of a weekly consumption of 2 to 4 serving of legumes, (chickpeas, lentils….) or in other words, the equivalent to a plate of legumes (60-80 grams raw)
8. A healthy diet consists of a weekly consumption of 3 to 7 servings or nuts. One serving equals a handful (20-30 grams).
9. A healthy diet consists of a daily consumption of 1.5 to 2 liters of water.

**SUPPLEMENTARY FILE 2 (S2)**

**BLOOD COLLECTION AND PROCESSING**

A 3-5 ml of venous blood was collected following standardized procedure from all participants by using disposable syringe. Samples were used for separation of plasma and serum. For the collection of plasma, blood samples were centrifuged for a period of 10 minutes at 1200 rmp. Blood was collected in Gel tubes and was allowed to clot at room temperature for the collection of serum for a period of 1hour before centrifugation. Serum and Plasma samples were processed for general blood chemistry (blood lipid profile, glucose and CRP) within 2 hours. Serum samples were stored at (−80°C) for shipment and further necessary analysis. Samples were shifted on dry ice to “Biology of Aging Laboratory, Singapore Immunology Network, Agency for Science Technology and Research, 8A Biomedical Grove, Singapore 138648, Singapore”. All analyses on chemokines and cytokines were performed using the facilities of these laboratories through a scientific research collaboration.

## **Blood Lipid Profile**

Total Cholesterol (TC), High Density Lipoprotein (HDL), Low Density Lipoprotein (LDL) and Triglycerides were measured by using previously established method. Blood samples were centrifuged for 10 minutes at 1200 rpm. Serum was separated and lipids were determined using commercially available working reagents for lipids (Excel Diagnostics, Pakistan). About 0.05 ml of serum sample was mixed to 1 ml of working reagent and was incubated at 37°C and analyzed using an auto-analyzer which works on principles of calorimetry.

The value of LDL cholesterol was estimated as LDL = TC - (HDL + TG)

## **Blood Glucose**

Glucose is oxidized by glucose oxidase with the synthesis of gluconic acid and hydrogen peroxide in this technique. The latter reacts with 4-aminophenazone (4AF) and phenol catalyzed by peroxidase to produce a red quinonimine with a maximum absorption wavelength of 505 nm. The color intensity is proportional to the concentration of glucose in the sample.

## **High Sensitivity C Reactive Protein**

High sensitivity C reactive protein concentration was measured by immune-turbidometry method by auto analyzer (Prestige 24i, Tokyo Boeki, Japan). In this method, the C-reactive protein reacts with the specific antibody forming insoluble immune-complexes. The turbidity produced by the immune-complexes is proportional to the CRP concentration in the sample and can be read with spectrophotometer.

**Cytokines/chemokine estimation**

Enzyme-Linked Immunosorbent Assay (ELISA) was used to determine the levels of sCD14 (Elabscience, Hubei, China). The procedure's details have already been reported (Alam *et al.,* 2019). Serum samples were placed in 96-well plates that had already been pre-coated with antibodies specific to the markers of interest. Enzyme-linked antibody conjugates were treated with bound markers. After properly washing the wells to eliminate any unbound antibodies, each well was filled with substrate solution. The reaction was terminated when color development was observed to be optimal, as per the manufacturer’s recommendations. The optical densities were measured at wavelengths indicated in the manufacturers’ protocol on the EnVision®2104 multimode micro-plate reader (Perkin Elmer, MA, U.S.A.). Data was acquired using xPonent® 4.0 (Luminex, TX, USA) software and analyzed with the Bioplex Manager™ 6.0 software (Bio-Rad Laboratories, Hercules, CA, U.S.A.).

**SUPPLEMENTARY FILE 3 (S3)**

**QUESTIONNAIRE: ADHERENCE TO NUTRITION EDUCATION**

|  | **Agree** | **Strongly Agree** | **Disagree** | **Strongly Disagree** |
| --- | --- | --- | --- | --- |
| I eat to eat according to the schedule recommended by the Dietitian. |  |  |  |  |
| I eat types/amount of foods according to the recommendation from the Dietitian. |  |  |  |  |
| I consume/would like to consume food that contains more vitamins and minerals. |  |  |  |  |
| I eat food that is rich in protein such as meat, eggs or soybean. |  |  |  |  |
| I eat fruits and vegetables every day as recommended by the Dietitian. |  |  |  |  |
| I weigh myself every day. |  |  |  |  |
| I do not want to follow the dietary recommendations from Dietitian because it is inconvenience for me. |  |  |  |  |
| I have been too busy with my work and I do not eat according to the recommended meal time. |  |  |  |  |
| I often consume sweet or sugary foods and drinks |  |  |  |  |
| I often eat fast food or fatty food (such as fries or animal innards). |  |  |  |  |
| I eat more than three major meals every day |  |  |  |  |
| I often consume salty food, such as dried fish |  |  |  |  |
| I often have snacks outside regular meal times |  |  |  |  |

**SUPPLEMENTARY FILE 4 (S4)**

**ADDITIONAL ANALYSIS ASSOCIATED WITH TABLE 3.**

**Table S3: Confidence Intervals (CI) of dietary PDGN scores**

| Variables | Intervention (*n* = 37) | | | Control (*n* = 37) | | |
| --- | --- | --- | --- | --- | --- | --- |
|  | T1 | T2 | T3 | T1 | T2 | T3 |
| Non-refined cereals and bread | 0.846 to 1.55 | 2.65 to 3.55 | 1.15 to 1.85 | 1.01 to 1.59 | 0.913 to 1.69 | 1.05 to 1.75 |
| Fruit | 1.35 to 2.45 | 3.72 to 5.08 | 2.11 to 2.89 | 1.77 to 1.83 | 1.55 to 2.25 | 1.11 to 1.89 |
| Vegetable | 3.22 to 4.58 | 3.63 to 5.37 | 4.02 to 4.98 | 3.22 to 4.58 | 2.55 to 3.25 | 3.35 to 4.05 |
| Legumes | 1.21 to 1.99 | 2.86 to 4.34 | 1.92 to 3.28 | 1.21 to 1.99 | 1.41 to 2.19 | 1.31 to 2.09 |
| Fish | 1.38 to 2.22 | 2.79 to 4.41 | 2.28 to 3.12 | 1.21 to 1.99 | 0.946 to 1.65 | 1.37 to 1.63 |
| Vegetable/Olive oil | 0.313 to 1.09 | 1.71 to 2.49 | 0.717 to 1.68 | 0.671 to 0.929 | 0.771 to 1.03 | 0.168 to 0.232 |
| Red Meat and meat products | 0.146 to 0.854 | 1.05 to 1.75 | 0.436 to 0.564 | 0.368 to 0.432 | 0.371 to 0.629 | 0.436 to 0.564 |
| Poultry | 0.146 to 0.854 | 2.18 to 3.02 | 1.01 to 1.39 | 0.436 to 0.564 | 0.671 to 0.929 | 0.503 to 0.697 |
| Full-fat dairy products | 0.746 to 1.45 | 2.92 to 4.28 | 1.07 to 1.13 | 1.17 to 1.43 | 0.813 to 1.59 | 1.07 to 1.33 |
| Sweets | 0.371 to 0.629 | 3.23 to 4.97 | 1.24 to 1.56 | 0.168 to 0.232 | 0.636 to 0.764 | 0.371 to 0.629 |
| Ghee | 0.403 to 0.597 | 3.02 to 4.38 | 1.35 to 2.05 | 0.671 to 0.929 | 0.636 to 0.764 | 0.371 to 0.629 |
| Total adherence to PDGN | 11.2 to 17.2 | 32.6 to 40.8 | 17.1 to 24.7 | 12.2 to 16.2 | 12 to 16 | 9.9 to 13.7 |

**ADDITIONAL ANALYSIS ASSOCIATED WITH TABLE 4**

The results of an ANOVA analysis show body weight had significant interaction with Time x Group (F (2. 57)=15.4; p<0.0001). Mean weight of intervention group by T2 was significantly lower compared with baseline (T1) (p=0.003) and that of control group unchanged (p>0.05). Mean weight of intervention and control groups by T3 was unchanged (p>0.05). Mean BMI of intervention group by T2 and T3 was significantly lower compared to baseline (p, for all trends (p, for all trends (<0.05) and for control group, the values were non-significantly lower (p, for all trends >0.05).

**Table S4: Confidence Intervals (CI) Values of Anthropometrics of Intervention and Control Groups**

| Variables | Intervention (n=30) | | | Control (n=30) | | |
| --- | --- | --- | --- | --- | --- | --- |
|  | T1 | T2 | T3 | T1 | T2 | T3 |
| Weight (Kg) | 70.3 to 78.1 | 69.1 to 76.7 | 70 to 77.6 | 67.7 to 75.3 | 66.7 to 74.1 | 67.3 to 74.9 |
| BMI | 1.1 to 3.92 | 23.2 to 26 | 23.5 to 26.3 | 23.2 to 25.8 | 22.8 to 25.4 | 23.2 to 25.8 |
| WC (cm) | 86.1 to 93.9 | 85.2 to 92.7 | 86.2 to 93.7 | 86.8 to 93.2 | 92.5 to 98.7 | 85 to 91.4 |
| %BF (cm) | 26.7 to 31.5 | 23 to 26.6 | 25.1 to 29.3 | 28.7 to 33.5 | 27.3 to 31.5 | 26.8 to 31.2 |

**ADDITIONAL ANALYSIS ASSOCIATED WITH TABLE 5**

The mean (SD) energy intake at T2 was significantly higher compared to T1 for intervention group. For control group, this was reversed. The protein intake at three time points remained unchanged both for intervention and control groups (P, for all trends>0.05). Compared to T1, intervention group had higher mean (SD) at T2, while the control group had lower mean (SD) at T2. Fiber intake remained stable without any significant changes (P, for all trends>0.05).

**Table S5: Confidence Intervals (CI) Values of nutrients intake of Intervention and Control Groups**

| Variables | **Intervention (n=30)** | | | **Control (n=30)** | | |
| --- | --- | --- | --- | --- | --- | --- |
|  | **T1** | **T2** | **T3** | **T1** | **T2** | **T3** |
| Energy (Kcal) | 1960 to 2150 | 2150 to 2230 | 2270 to 2370 | 2200 to 2400 | 2140 to 2240 | 2350 to 2430 |
| Carbohydrates (g) | 284 to 319 | 322 to 343 | 339 to 357 | 338 to 369 | 336 to 355 | 304 to 378 |
| Total Protein (g) | 67.7 to 74.7 | 68.1 to 72.7 | 69.9 to 74.3 | 67 to 75.6 | 69.7 to 75.3 | 69.6 to 75 |
| Fats (g) | 58.6 to 65.9 | 60.5 to 68.3 | 68.1 to 74.5 | 61.3 to 72.9 | 61.3 to 72.9 | 69.1 to 74.7 |
| Fiber (g) | 10.4 to 11.8 | 10.1 to 11.3 | 9.93 to 11.1 | 9.29 to 12.5 | 10.2 to 11.6 | 9.56 to 10.8 |

**ADDITIONAL ANALYSIS ASSOCIATED WITH TABLE 6**

The two-way ANOVA (Time x Group) for blood glucose showed no significant effects for Time x Group interactions (F (2, 116) = 0.9; P=0.390) or for group (F (1, 116) =0.2; P=0.6). However, there was a significant time effect (F (2,1 16) =19.1; P<0.001). Blood cholesterol showed significant effects for Time x Group interaction (F (2,116) =3.5; P=0.033) or for time effect (F (1, 116) =1.35; P=0.005). However, there was a non-significant group effect (F (2,116) =1.359.1; P=0.244). Plasma Na showed significant effects for Time x Group interaction (F (2,116) =31.5; P<0.0001) or for time effect (F (1,116) = 31.3; P<0.0001) and for group effect (F (2,116) = 41.3; P<0.0001). CRP showed significant effects only for time effect (F (1,116) = 2.9; P=0.018) but non-significant effect for Time x Group interaction (F (2,116) =31.5; P<0.048) or group effect (F (2,116) =0.01; 41.3; P=0.93). The two-way ANOVA (Time x Group) for CRP showed significant effects only for time effect (F (1,116) =2.9; P=0.018) but non-significant effect for Time x Group interaction (F (2,116) =31.5; P<0.048) or group effect (F (2,116) =0.01; 41.3; P=0.93). Systolic BP showed significant effects for Time x Group interaction (F (2,116) =431.5; P<0.0001) or for time effect (F (1,116) =602.3; P<0.0001) and for group effect (F (2,116) =641.3; P<0.0001). Similarly, diastolic BP showed significant effects for Time x Group interaction (F (2,116) =29.1; P<0.0001) or for time effect (F (1,116) =13.9; P<0.0001) and for group effect (F (2,116) =24.6; P<0.0001).

**Table S6: Confidence Intervals (CI) Values of Bio-chemicals and Diastolic and Systolic Blood Pressure**

| **Variables** | **Intervention (n=30)** | | | **Control (n=30)** | | |
| --- | --- | --- | --- | --- | --- | --- |
|  | **T1** | **T2** | **T3** | **T1** | **T2** | **T3** |
| Glucose (mg/dl) | 18 to 129 | 93.4 to 104 | 100 to 110 | 113 to 129 | 96.4 to 106 | 90.1 to 110 |
| Cholesterol (mg/dl) | 126 to 173 | 57.9 to 111 | 110 to 190 | 104 to 163 | 80.7 to 135 | 68.1 to 125 |
| HDL (mg/dl) | 32.6 to 39.2 | 30 to 38.8 | 29.1 to 37.6 | 27.6 to 34.2 | 29.4 to 35.4 | 26.4 to 34.4 |
| LDL (mg/dl) | 86 to 139 | 38.6 to 61.6 | 96.2 to 137 | 77.3 to 93.9 | 63.2 to 87.8 | 65.8 to 88 |
| Sodium (mg) | 3.08 to 3.32 | 2.8 to 3.2 | 2.91 to 3.49 | 2.82 to 2.98 | 2.61 to 2.79 | 2.88 to 3.12 |
| CRP (pg/ml) | 3 to 3.8 | 2.45 to 3.35 | 4.71 to 6.22 | 3.36 to 4.84 | 2.96 to 4.24 | 2.52 to 4.08 |
| Diastolic BP (mmHg) | 80 to 81 | 77.4 to 79.2 | 78.3 to 81.3 | 80 to 81.6 | 78.4 to 80 | 79.9 to 80.9 |
| Systolic BP (mmHg) | 120 to 126 | 114 to 118 | 110 to 114 | 121 to 126 | 113 to 117 | 121 to 124 |

**ADDITIONAL ANALYSIS ASSOCIATED WITH TABLE 7**

**Table 7S: Confidence Intervals (CI) Values of cytokines/chemokines**

| Variables | Intervention (n=30) | | | Control (n=30) | | |
| --- | --- | --- | --- | --- | --- | --- |
|  | T1 | T2 | T3 | T1 | T2 | T3 |
| IL-2 (pg/ml) | 1.19 to 1.55 | 1.61 to 2.37 | 1.37 to 1.93 | 1.25 to 1.95 | 1.45 to 2.23 | 1.31 to 1.93 |
| IL-7 (pg/ml) | 3.66 to 6.74 | 4.65 to 6.15 | 5.05 to 6.55 | 3.6 to 6.6 | 4.08 to 5.72 | 3.14 to 6.86 |
| TNF-α (pg/ml) | 24.9 to 48.1 | 53.3 to 97.1 | 37.2 to 67 | 42.8 to 45.4 | 50 to 62.2 | 50.2 to 59.6 |
| Resistin (pg/ml) | 3020 to 4290 | 2880 to 5080 | 1840 to 5740 | 2930 to 4380 | 3460 to 4520 | 3130 to 4300 |
| IL-1 RA (pg/ml) | 49.9 to 112 | 94.9 to 186 | 67.7 to 117 | 76 to 146 | 89.3 to 145 | 102 to 150 |
| IL-4 (pg/ml) | 36.2 to 56.6 | 42.2 to 66.4 | 40.3 to 64.9 | 32.7 to 55.9 | 45.4 to 66.8 | 43.5 to 66.3 |
| IL-17 A (pg/ml) | 4.07 to 6.33 | 4.24 to 6.66 | 4.71 to 7.07 | 4.27 to 6.37 | 3.22 to 6.08 | 4.95 to 6.71 |
| sCD40 (pg/ml) | 17.6 to 23.3 | 16.7 to 24 | 9.29 to 17.7 | 40.9 to 47.2 | 51.9 to 60.1 | 51.2 to 58 |
| TGF-α (pg/ml) | 5.64 to 8 | 5.93 to 9.89 | 6.41 to 9.25 | 5.37 to 8.79 | 5.75 to 7.93 | 5.61 to 8.25 |

**References**

Alam, I., R. Gul, J. Chong, C.T.Y. Tan, H.X. Chin, G. Wong, R. Doggui and A. Larbi. 2019. Recurrent circadian fasting (RCF) improves blood pressure, biomarkers of cardiometabolic risk and regulates inflammation in men. Journal of translational medicine. 17: 272.
